# The deep and slow breathing characterizing rest favors brain respiratory-drive

**DOI:** 10.1038/s41598-021-86525-3

**Published:** 2021-03-29

**Authors:** Baptiste Girin, Maxime Juventin, Samuel Garcia, Laura Lefèvre, Corine Amat, Nicolas Fourcaud-Trocmé, Nathalie Buonviso

**Affiliations:** 1 Lyon Neuroscience Research Center (CRNL), Inserm U 1028, CNRS UMR 5292, University Lyon 1, 69675 Bron, France; 2 Present Address: Medical Research Council Brain Network Dynamics Unit, Nuffield Department of Clinical Neurosciences, University of Oxford, Oxford, OX1 3TH UK

**Keywords:** Neural circuits, Neuroscience, Olfactory system

## Abstract

A respiration-locked activity in the olfactory brain, mainly originating in the mechano-sensitivity of olfactory sensory neurons to air pressure, propagates from the olfactory bulb to the rest of the brain. Interestingly, changes in nasal airflow rate result in reorganization of olfactory bulb response. By leveraging spontaneous variations of respiratory dynamics during natural conditions, we investigated whether respiratory drive also varies with nasal airflow movements. We analyzed local field potential activity relative to respiratory signal in various brain regions during waking and sleep states. We found that respiration regime was state-specific, and that quiet waking was the only vigilance state during which all the recorded structures can be respiration-driven whatever the respiratory frequency. Using CO_2_-enriched air to alter respiratory regime associated to each state and a respiratory cycle based analysis, we evidenced that the large and strong brain drive observed during quiet waking was related to an optimal trade-off between depth and duration of inspiration in the respiratory pattern, characterizing this specific state. These results show for the first time that changes in respiration regime affect cortical dynamics and that the respiratory regime associated with rest is optimal for respiration to drive the brain.

## Introduction

Transferring information across distant brain regions is essential for the expression of specific behaviors. The communication through coherence hypothesis^1^ recently sets the respiratory rhythm as a global rhythm, potentially influencing behavior by modulating cortical neurodynamics^2, 3^. Indeed, number of studies have described breathing as modulating complex behaviors. In animals, respiration notably interacts with vocalizations, whisking and licking^4, 5^. In humans, a clear relationship exists between breathing and cognitive processing^2^. Notably, respiration modulates olfactory memory consolidation^6^ and cognitive performance during retrieval^7^. Pain processing is gated during exhalation^8^. In experiments with forced breathing, the gripping force is greater during exhalation than during inhalation^9^. The recognition of facial fear expression is faster if the stimulus is presented during the inspiratory phase^10^. Even when no instructions for inspiration are given, subjects inhale spontaneously at the beginning of a cognitive task and these inhalations induce changes in brain network functional connectivity^11^. Importantly, this last study also emphasized the involvement of nasal respiration in improving performance in a visuo-spatial task.

Respiration also modulates ongoing activity in diverse cerebral regions both in rodents^12–17^ and humans^10, 18^. Such a respiratory drive surely depends on nasal respiration as it is suppressed when nasal airflow is by-passed. In rodents, respiration-locked activity is eliminated in the barrel cortex when nasal airflow is stopped after olfactory bulbectomy^12^; in hippocampus, respiration-related slow rhythm is suppressed under tracheotomy^19, 20^ and influence of respiration on sharp wave ripples occurrence is eliminated when olfactory bulb (OB) activity is inhibited ^15^. In human, oscillatory power in olfactory cortex and limbic structures dissipates when subjects breathe through the mouth^10^. Even if the involvement of a central component cannot be excluded^21^, the main origin of this respiratory drive during nasal breathing is likely to be found in the olfactory receptor cells mechano-sensitivity to pressure changes in the nasal cavity during successive inspirations and expirations^22^.

A respiration-locked activity propagates from the olfactory epithelium to the OB where it has been extensively described. In brief, respiration has been shown to modulate OB local field potential (LFP) fast oscillations^23^, and both unitary discharges^24–26^ and membrane potential of principal neurons^27, 28^. Interestingly, changes in airflow rate are sufficient to substantially reorganize the OB response: cellular odor-evoked activities and LFP oscillations are strongly modified by nasal flow rate^29–32^. A low/high flow rate favors a beta/gamma regime in the OB, respectively^31^. Nasal airflow rate, but not respiratory frequency, is a key factor that regulates olfactory sensitivity of the OB glomeruli as shown by calcium imaging combined with an artificial sniffing setup^29^.

Yet, nasal airflow varies in both flow rate and frequency according to respiration dynamics. Therefore, if the respiratory drive of the brain originates in nasal airflow movements, then it should vary with respiration dynamics that occur spontaneously during natural conditions. We took advantage of the spontaneous variations of respiration dynamics during waking and sleep states to explore respiratory drive in various brain regions: olfactory bulb (OB), piriform (AP), prefrontal (PFC), somatosensory (S1) and visual (V1) cortices, dorsal CA1 (CA1) and dentate gyrus (DG) hippocampal areas. We analyzed their LFP activity relative to respiratory signal. Importantly, we did not restrict our study to epochs of respiratory steady state as chosen by others^17^ but instead included signals from full waking and sleep periods, thus encompassing the wide range of respiratory frequencies expressed by a freely moving animal. This reveals that respiratory regime was state-specific, and that quiet waking was the only vigilance state where both olfactory and non-olfactory structures can be respiration-coupled whatever the respiration frequency (0.8–5 Hz) expressed during this state. We used a CO_2_-enriched air in the plethysmograph to alter the respiratory regime associated with each state. Using a respiratory cycle based analysis, we evidenced that the large and strong brain entrainment during quiet waking was related to its specific respiration regime consisting in an optimal trade-off between depth and duration of inspiration. These results show for the first time that changes in respiratory regime alter the cortical dynamics and that the respiratory regime observed during rest is optimal for respiration to drive the brain.

## Results

**Figure 1:**
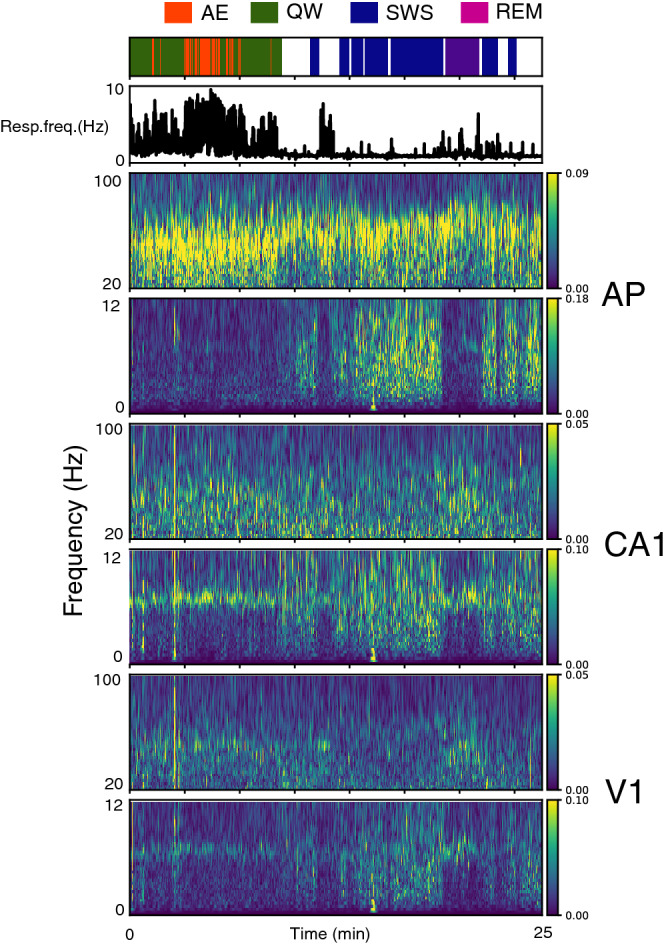
Example of a 25 min long recording in anterior piriform cortex (AP), hippocampus CA1 and primary visual cortex (V1) regions. Top panel: states scoring with active exploration (AE, red), quiet awaking (QW, green), slow-wave sleep (SWS, blue), and rapid-eye movements sleep (REM, pink). Periods not scored (not all criteria met, see “Methods”) appear in white. Second panel: Instantaneous respiratory frequency (in Hz). Other panels from top to bottom: For each structure (AP, CA1, V1), time–frequency representation of LFP in the low (0.5–12 Hz) and high (20–100 Hz) frequency range. Warm colors indicate high power.

**Figure 2:**
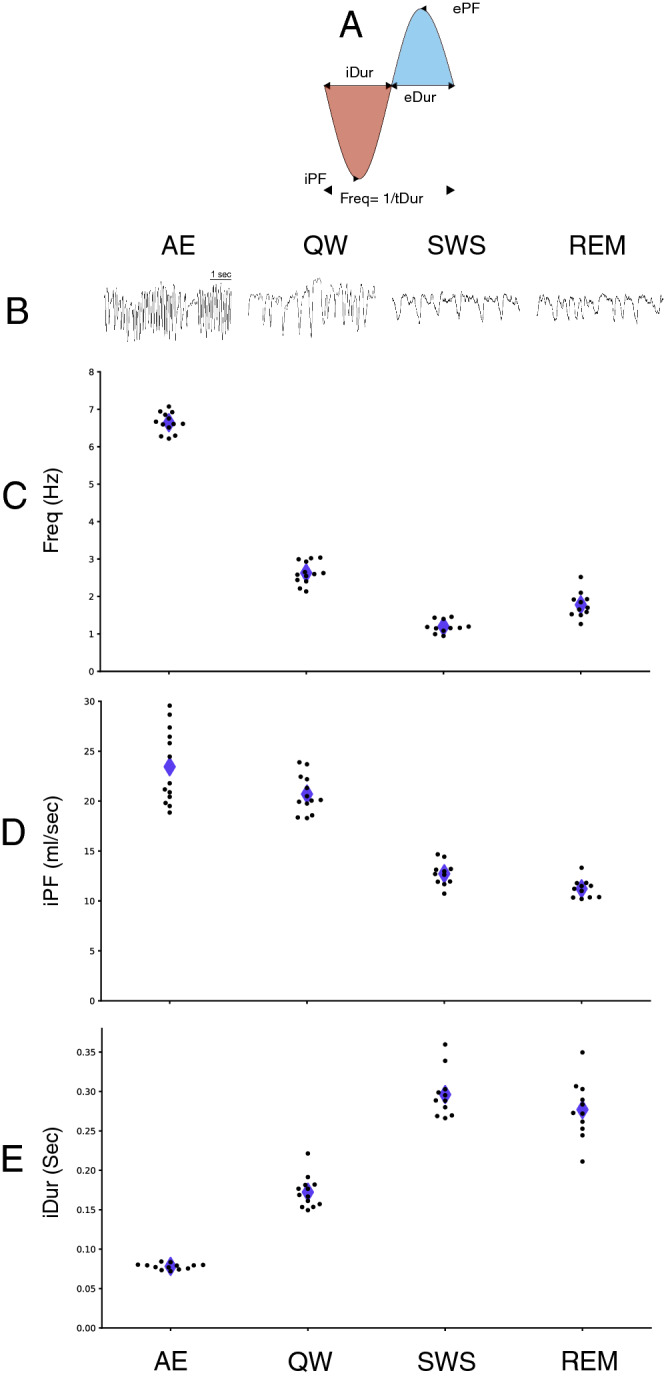
Respiration regimes associated with brain states. (**A)** Schematic representation of measured respiratory features. (**B)** Examples of respiration raw traces during active exploration (AE), quiet waking (QW), slow-wave sleep (SWS) and rapid eye movements sleep (REM). (**C–E**) Scatter plot of respiration features measured during each brain state (average per rat): frequency (Freq, **C**), inspiration peak flow rate (iPF, **D**), and inspiration duration (iDur, **E**). Repeated measure ANOVA tests with the 11 rats recorded in all states show significant effects of state on all respiratory features (see main text for details). Black dots: individual values per animal; Purple diamonds: means.

Respiration signal, recorded via a whole-body plethysmograph, and LFPs were collected from 13 rats during active exploration (AE), quiet waking (QW), slow-wave sleep (SWS) and rapid-eye movements (REM) episodes (Fig. 1; see “Methods” section for state scoring). Typically, respiratory frequency was higher during awake states and highly variable, whereas it was low and more stable during sleep states. LFP activities varied both in the low (0.5–10 Hz) and high (20–100 Hz) frequency bands. A major innovation of our study is that we analyzed data from the wide range of 0-10 Hz respiration frequencies, i.e. neither restricting to epochs where respiration frequency did not overlap with theta frequency nor focusing on stationary periods, as proceeded in a similar study^17^. This allowed us to track faithfully respiratory brain entrainment under non-stationary respiration regimes. We first explored to what extent respiratory regime varied with behavioral state by analyzing the following features (Fig. 2A):

– instantaneous respiratory frequency (Freq, in Hz), extracted from inhalation and exhalation durations (iDur, eDur).

– inspiration and expiration peak flow rate (iPF, ePF, respectively, in mL s^−1^), representing the maximal flow rate.

Representative raw traces (Fig. 2B) illustrate the diversity of respiratory patterns between the different states, which was confirmed at the group level (Fig. 2C-E). All respiration features significantly varied between states (repeated measures ANOVA test: for Freq: *F*_3,30_ = 65.0, p < 0.001; for iPF: *F*_3,30_ = 114.4, p < 0.001; for iDur: *F*_3,30_ = 344.1, p < 0.001. Post-hoc paired t-test with Holm correction showed highly significant differences between all state pairs, p < 0.001, except p = 0.019 between SWS and REM for iDur and p = 0.073 between SWS and REM for iPF. For all tests N = 11 because 2 rats which have not been recorded during SWS and REM states have been excluded, but their data points in awake states are still plotted in Fig. 2C-E). Freq was particularly higher during AE (mean 6.58 Hz, SD 0.24 Hz, Fig. 2C) compared to QW (mean 2.56 Hz, SD 0.27 Hz) as sniffing activity was predominant and characterized by i) high iPF even if highly variable (AE: mean 23.8 mL/s, SD 3.9 mL/s; compared to QW: mean 20.8 mL/s, SD 2.0 mL/s, Fig. 2D) and high ePF (AE: mean 19.0 mL/s, SD 2.5 mL/s; compared to QW: mean 14.0 mL/s, SD 1.5 mL/s, not shown) and ii) very short iDur (AE: mean 0.08 s, SD 0.004 s; compared to QW: mean 0.18 s, SD 0.02 s, Fig. 2E) and very short eDur (AE mean: 0.097 s, SD 0.006 s; compared to QW: mean 0.31 s, SD: 0.06 s, not shown). iPF was much higher for waking states (AE and QW, see above for values) than for sleep ones (SWS: mean 12.7 mL/s, SD 1.2 mL/s; and REM: mean 11.2 mL/s, SD 0.9 mL/s). Similarly, iDur was shorter for waking states (AE and QW, see above for values) than for sleep ones (SWS: mean 0.30 s, SD 0.03 s; and REM: mean 0.28 s, SD 0.04 s). Then a slow Freq, a low iPF, and a long iDur characterized sleep states. Similar observations and statistical differences were made on expiration features (not shown). To summarize, AE was characterized by a high iPF and a short iDur (related to sniffing activity), whereas both sleep states exhibited a low iPF and a long iDur. QW regime had distinctive features with both iPF and iDur in the mid-range.

**Figure 3:**
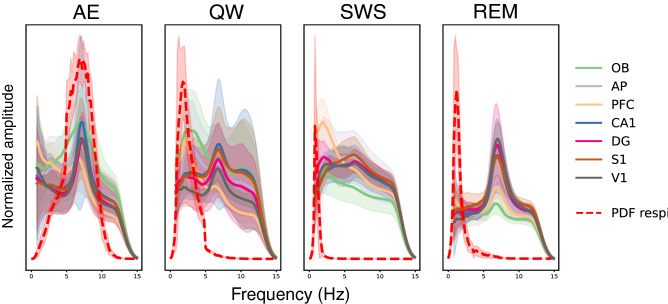
Normalized power spectral density of LFP and probability density function (PDF) of respiration frequency during the four brain states (AE active exploration, QW quiet waking, SWS slow-wave sleep, REM rapid eye movements sleep). Normalized spectra of LFP signals recorded in each structure (solid lines) were averaged across rats. Shaded areas display the standard deviations. The probability density function (PDF) of respiration frequencies (dotted red lines) were average across rats, shaded areas display the standard deviations. See sample size (number of rats and sites per state) in Table 1. See “Methods” section for spectrum normalization procedure but note that relative amplitude of spectra across states has been preserved.

Then, we investigated to what extent brain respiratory-drive, as described by others^3^, varied with brain state. We thus tracked respiratory frequency in the LFP recordings of each structure, during each state. Visual inspection of power spectra (Fig. 3) showed no clear overlap between respiration and LFP oscillations frequencies, except during AE where the animals actively explored the environment. Nevertheless, it is difficult in that case to disentangle sniffing-related activity (6.58 Hz, SD 0.24 Hz) from the prominent exploration-related theta rhythm (about 7 Hz, as measured in dorsal CA1 during REM^17^). We observed that theta rhythm (6-8 Hz range, according to peaks in spectra, see Fig. 3) was a major frequency during all states, except during SWS where delta activity (0–5 Hz) dominated. During QW, in addition to a clear theta rhythm in all structures except OB (Fig. 3, green line), spectral analysis evidenced a high power in the delta band (0–5 Hz), in all structures, partly overlapping with respiratory frequencies. During SWS, while respiratory frequency was very slow and constant around 1 Hz, LFP power was high in the whole delta band. Similarly, during REM state, respiration and LFP frequencies poorly overlapped: while LFP power peaked in the theta range (around 7 Hz), respiration frequencies lied in a much lower range (0.5–4 Hz). To summarize, these results suggests that brain respiratory-drive probably varies with brain state even if we could not observe a clear overlapping between respiratory and LFP frequencies.

**Table 1:** Coordinates from bregma (mm) and number of rats (n) in each condition (air and CO_2_).

Brain structure	AP	ML	DV	n air (AE and QW)	n air (SWS and REM)	n CO_2_ (all states)
Olfactory bulb, OB	5.5^a^	1.5	2	9	7	2
Anterior piriform cortex, AP	2.76	3.5	~ 6	13	11	5
Dorsal hippocampus CA1, CA1	− 2.92	2	~ 3.5	13	11	5
Dentate gyrus, DG	− 4.8	2.8	~ 3.2	12	10	5
Primary visual cortex, V1	− 5.28	4.2	~ 1.2	9	7	5
Primary somatosensory barrel cortex, S1	− 2.4	5	~ 2	12	10	5
Prefrontal cortex, PFC	3	0.8	~ 3.5	5	5	5

Note that in air condition, we distinguished awake and sleep states because 2 rats were not recorded in sleep states.

^a^From naso-frontal suture.

**Figure 4:**
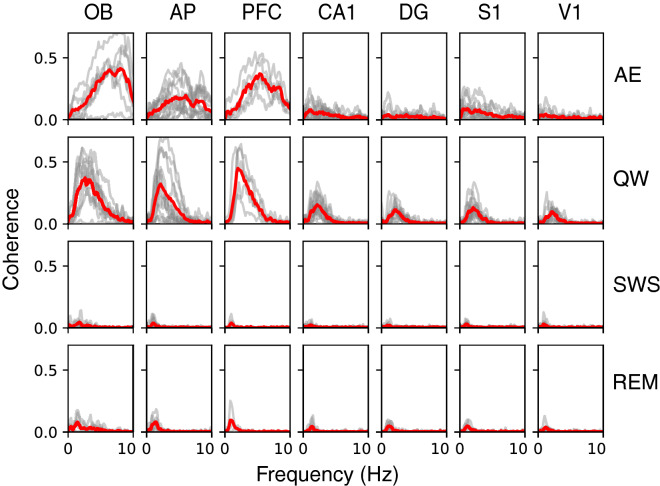
Coherence spectra between LFP and Respiratory signals during the four brain states (AE active exploration, QW quiet waking, SWS slow-wave sleep, REM rapid eye movements sleep), in all structures, in the 0.5–10 Hz range. Results obtained from simultaneous recordings in each rat (grey lines) and averaged across animals (red lines). See sample size (number of rats) in Table 1 and p values in Supplementary Table S1.

To go further, we then performed coherence analyses between LFP signals for all the recorded brain regions and respiration in the low frequency band (0.5–10 Hz) during the four states (Fig. 4). As described in “Methods” section, peak coherence values for actual data were compared to surrogates data. Actual and surrogates distributions significantly differed in all recorded regions and all states (see Supplementary Table S1), suggesting that all the recorded areas were respiration-coupled whatever the brain state. However, visual inspection of coherence spectra (Fig. 4) and actual values (Supplementary Table S1) relativized this conclusion since it is obvious that coherence spectra are particularly flat during SWS and REM, whatever the structure. On the contrary, coherence appeared more clearly in waking states (AE and QW, Fig. 4). During AE epochs, the respiration-LFP coherence was high (0.2–0.7) for Freq > 5 Hz and only in olfactory structures (OB, AP) and PFC. It is noteworthy that QW state was the only state during which coherence spectra appeared not flat in all the recorded areas. During SWS and REM, although power spectra exhibited a high power in the low frequency range (see Figs. 1, 3), the coherence with respiration, even if significant, was very weak (10 times smaller than during QW). To summarize, when analyzing respiration-LFP coherence over a large range of respiratory frequencies, we observed that respiratory drive of brain areas varied with vigilance state, QW state being the state where LFP-respiration coherence is the highest in all the structures.

**Figure 5:**
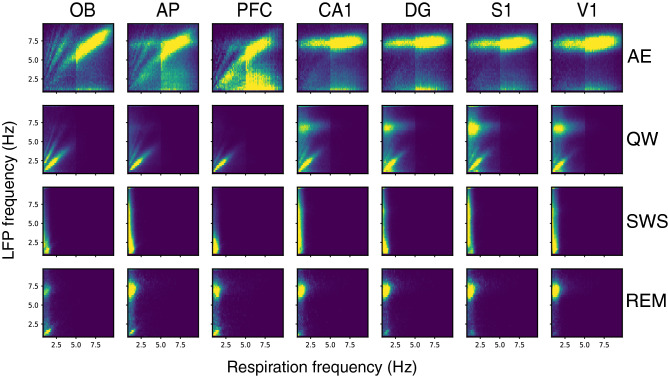
Covariation between LFP and respiration frequencies. Covariation maps, averaged across rats, obtained from LFP signals recorded under ambient air in OB, AP, PFC, CA1, DG, S1, and V1. Y-axis represents LFP frequency and X-axis respiratory frequency. The maps are 2-dimensional distributions of joined LFP-respiration frequencies across time (see methods for details), normalized so that the total sum is 1. Point density is represented on a color scale ranging from blue (0) to yellow (map maximum) as the point density increases. For each structure and state, see sample size (number of rats in the average) in Table 1.

Then, we searched in which state respiratory frequency could dominate in the LFP signal. We computed covariation maps (2-dimensional distribution of joined LFP-respiration frequencies across time) for all states and recorded structures, as described in “Methods”, focusing on the 0.5–10 Hz frequency range (Fig. 5). This analysis beneficially complements coherence analysis since it allows tracking instantaneous synchrony in the data. A highlighted diagonal in the covariation maps indicates that LFP predominantly oscillated at the respiratory frequency. Significance of these data has been assessed by comparing, for each rat, the coupling index measured in air condition with coupling indices of 100 surrogate maps obtained by shuffling LFP frequencies across time (see Supplementary Fig. S1, Supplementary Table S2, and methods section). During AE, where rats sniffed in the 5–10 Hz range, the predominant LFP frequency clearly followed respiration in olfactory structures and in PFC (see the highlighted diagonal in OB, AP, PFC and Supplementary Table S2). Oppositely, covariation maps in the other structures showed a uniform theta band activity whatever the respiration frequency (see the highlighted horizontal band in CA1, DG, S1, V1) indicating that these areas maintained an activity in the theta range whatever the respiration frequency. During QW, respiration frequency became predominant in the LFPs of all recorded structures as assessed by the highlighted diagonal in the 1–4 Hz range and by the comparison between actual and surrogate coupling index values (Supplementary Fig. S1B and Supplementary Table S2). In four structures (S1, CA1, V1, DG) respiration frequency co-dominated with theta nevertheless (Fig. 5, see the yellow spot around 7 Hz). During SWS, the range of respiration frequencies was narrow (see the low variation in Fig. 2C1) so that covariation analysis was not relevant for this state. During REM, LFP predominant frequency was theta and, even if covariation maps showed a highlighted diagonal in OB, AP, PFC and DG (Supplementary Fig. S1A), comparison between actual and surrogate data (Supplementary Fig. S1B) revealed no difference between conditions. These results revealed that LFP mainly oscillated at respiratory frequencies in all the recorded structures only during QW state.

**Figure 6:**
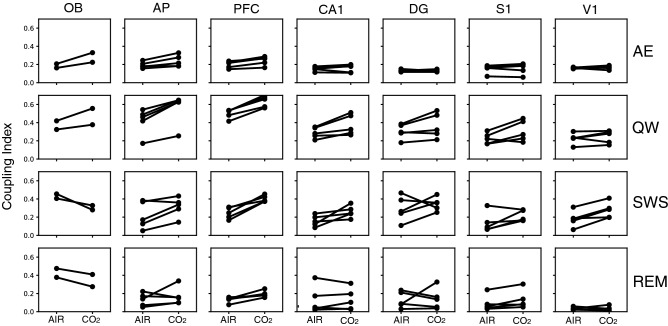
Comparison of respiration-LFP coupling between AIR and CO_2_ conditions. For each state (lines), each structure (columns), and each rat (black lines), the coupling index is plotted in AIR and CO_2_ conditions.* AE* active exploration, *QW* quiet waking, *SWS* slow-wave sleep, *REM* rapid eye movements sleep.

We then explored if the specific respiratory drive of the brain observed during QW could be more related to the specificity of respiration features (frequency and/or peak flow rate) or to that of the QW state itself. To disentangle state *vs* respiration regime effect, we altered the respiration regime associated with each state using a 5% CO_2_-enriched air in the plethysmograph (n = 5 rats). CO_2_ enrichment effectively strongly modified respiratory regime, as summarized in Supplementary Figure S2. Indeed, with addition of CO_2_, Freq significantly varied during AE, QW, and SWS (Supplementary Fig. S2 top panel), iPF greatly increased during all the four states (Supplementary Fig. S2, middle panel), and iDur strongly decreased during SWS and REM (Supplementary Fig. S2, bottom panel). Under such conditions, LFP-respiration coherence was slightly improved (Supplementary Fig. S3), particularly during QW state, in all structures (compare Fig. 4 and Supplementary Fig. S3). Then, covariation maps were also computed for both ambient AIR and CO_2_ conditions (n = 5, Supplementary Fig. S4). Under CO_2_ condition, covariation maps revealed subtle changes (Supplementary Fig. S4B) that we evidenced by comparing the coupling index between respiration and the predominant LFP frequency in the different structures, during the four states (Fig. 6). As explained in “Methods” section, the coupling index represents the sum of density along the diagonal of the covariation map and varies between 0 and 1 (with 1 being the highest coupling value). Individual data are shown in Fig. 6. The population analysis showed a significant interaction between the condition (AIR or CO_2_) and animal state (F_3, 184.5_ = 3.53, p = 0.016) but not between condition and structure (F_6, 184.5_ = 1.70, p = 0.12). We thus looked at results at the animal state level. During AE, the coupling index did not show much changes between ambient AIR and CO_2_ conditions (AIR 0.16 *vs* CO_2_ 0.18; T-test, t = -1.49, df = 185, p = 0.18) even if olfactory areas and PFC showed a slight increase of coupling index in all the rats. This probably indicates that, even if iPF increased significantly under CO_2_ (see Supplementary Fig. S2), LFP frequency in these areas was still dominated by theta rhythm. During QW, the coupling index, already high under AIR condition, markedly increased (AIR 0.33 *vs* CO_2_ 0.42; T-test, t = -4.75, df = 185, p < 0.0001), although it increased very modestly in V1. During SWS, even if delta band remained the major frequency, the coupling index increased under CO_2_ in most rats and most structures suggesting that respiratory frequency became more predominant under CO_2_ compared to AIR condition (AIR 0.22 *vs* CO2 0.30; T-test, t = -3.36, df = 185, p = 0.0019). We even observed highlighted diagonal on a short range of respiration frequencies (white arrows, Supplementary Fig. S4B). During REM, while covariation maps exhibited a clearer and more extended highlighted diagonal (white arrows, Supplementary Fig. S4B), the coupling index in Fig. 6 appeared generally comparable between CO_2_ and AIR condition (AIR 0.13 *vs* CO_2_ 0.14; T-test, t = -0.56, df = 185, p = 0.58) except few exceptions (1 rat in AP, all rats in PFC, 1 rat in DG, 2 rats in S1). This is probably because, again here, theta rhythm still dominated, as observed in the covariation maps of all structures except OB (Supplementary Fig. S4). Overall, these results suggest that the strong respiratory brain drive during QW was not related to the neuronal activity characteristics of this peculiar brain state but rather to the respiratory regime associated with this state. Indeed, when extending the respiratory regime of sleep states, via CO_2_-enriched air, to a wider range of frequencies and higher flow rates, respiration frequency became more predominant in LFP signals relative to ambient air condition.

**Figure 7:**
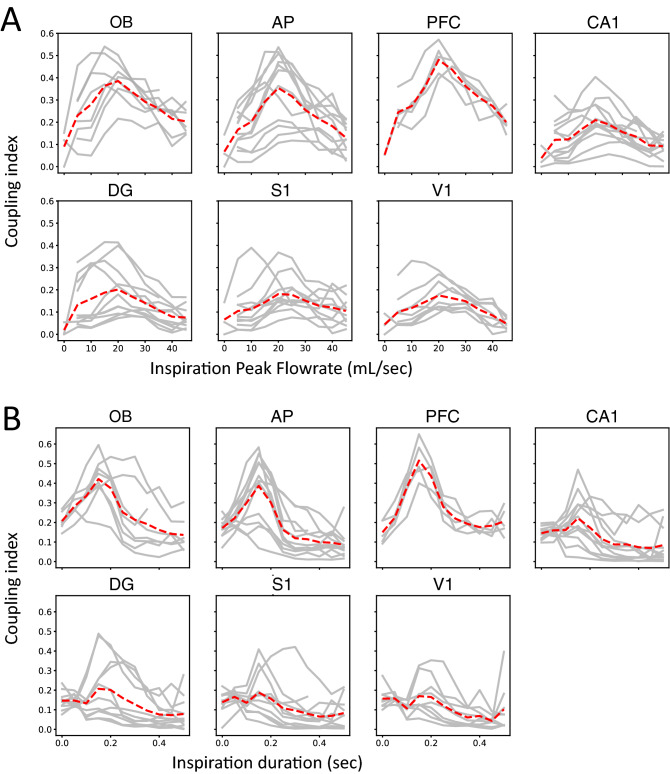
Coupling index as a function of inspiration peak flow rate iPF (**A**) and inspiration duration iDur (**B**). Data are presented individually for each animal (solid lines, see Table 1 for sample size), and averaged across animals (dotted red lines). The inverted U-shape of coupling index as a function of iPF and iDur was assessed by comparing two generalized mixed linear-models either with or without the quadratic term (see “Methods” for details). The comparison showed that the iPF quadratic term was significant for all areas (Wald test, p < 0.02, see text for details) and that the iDur quadratic term was significant for some areas (Wald test, PFC: p = 0.01, and trends for AP and OB, p < 0.1, see text for details).

Since CO_2_ condition increased both peak flow rate and frequency, the next step was to decipher which of the respiration features was decisive in entraining brain structures to respiration. We focused on two respiration features determining the depth and frequency of respiration: inspiration peak flow rate (iPF) and inspiration duration (iDur). We thus performed a coupling analysis between respiration and LFP frequencies, independently of the brain state, segregating respiratory cycles according to their values of iPF (Fig. 7A) and iDur (Fig. 7B). Both individual and averaged coupling index curves (sample size, see Table 1) followed an inverted-U shape curve: close to zero for low iPF values, it reached a maximum for iPF values around 20 mL s^−1^, then decreased for higher iPF values. This shape was similar for all structures, but with different amplitudes. Indeed, the respiration—LFP coupling as a function of iPF was very strong in olfactory structures and PFC, less so in other areas, but nevertheless still significant (quadratic term significance—i.e. existence of a local maximum—was assessed by comparing generalized mixed-effect linear models with Wald test (df = 1): OB: χ^2^ = 7.88, p = 0.005; CA1: χ^2^ = 6.67, p = 0.01; PFC: χ^2^ = 10.02, p = 0.0015; DG: χ^2^ = 11.03, p = 0.0009; AP: χ^2^ = 12.27, p = 0.0004; S1: χ^2^ = 7.87, p = 0.005; V1: χ^2^ = 7.88, p = 0.005). Similar results were obtained when computing the coupling index as a function of iDur (Fig. 7B). As for iPF, the coupling index indeed evolved following an inverted U-shape curve, optimal for a range of iDur values around 0.15 s (quadratic term was significant for PFC: χ^2^ = 6.34, p = 0.01; and there were trends for AP: χ^2^ = 3.09, p = 0.08; and OB: χ^2^ = 2.8, p = 0.09). To explain the inverted-U shapes of the coupling index curves, we have to consider iPF and iDur simultaneously. In Fig. 7A, the highest values of iPF correspond to the sniffing cycles during AE, which present the shortest iDur. The decreasing phase which followed (Fig. 7B, iDur > 0.3 s) for long iDur values correspond to the sleep states breathing cycles, which also have small iPF values.

Finally, these results show that the best coupling between respiration and LFP frequencies is achieved for inspiration peak flow rate in the range of 20–30 mL/s combined with 0.1–0.2 s inspiration duration. This trade-off between iPF and iDur corresponds to the specific features of respiratory regime associated with the QW state.

## Discussion

We studied how respiration could drive brain areas during spontaneous variations of respiratory regime related to four different behavioral states: active exploration, quiet waking, slow-wave sleep and paradoxical sleep. In addition to confirming previous observations that LFPs in different brain structures can actually be respiration-coupled^12–17^, we unveiled new fundamental findings: (1) a large-scale respiratory drive is a specific characteristic of the quiet waking state, (2) this specificity is due to the respiratory regime associated with this state and not to the brain state itself, (3) an optimal respiratory regime for large brain entrainment is a deep and long inhalation (peak flow rate in the range of 20–30 mL/s and duration in the range of 0.1–0.2 s).

We described for the first time that, even if each of the recorded structures can be occasionally entrained by respiration as reported by others^17, 33^, a global respiratory drive of the brain only emerged during quiet waking state. A respiratory drive in different brain areas with regard to the brain state has been previously described. However, none of them reported this specificity of the quiet waking state. Indeed, while one study focused on rest periods (similar to the QW state in our study) and exploration (similar to the AE state in our study) to investigate how gamma band activity in distant areas phase-locked to respiratory activity of the olfactory bulb^33^, an EEG study in the cat described the respiratory slow wave in the waking state but did not differentiate rest from exploration^34^. Respiratory drive in different brain areas was also explored but restricted to exploration periods recorded in the animal home cage and REM sleep epochs recorded in a plethysmograph^3^. Furthermore, because in both states theta oscillations and respiration may overlap in frequency, they also chose to analyze only epochs where respiration frequency and theta frequency were not overlapping. We took a different approach by exploring LFP-respiration covariation whatever the brain state, and including all respiratory frequencies. Finally, we have been the first to explore respiratory drive simultaneously in a large brain network, during four brain states, and during the whole duration of recordings. This strategy allowed us to highlight the specificity of the quiet waking state.

We also provided evidence that the global respiratory drive observed during quiet waking is not related to the state itself but rather to the respiratory regime associated. This demonstration has been possible because, using a CO_2_-enriched air in the plethysmograph, we modified the respiratory regimes associated with the different states, thus extending the range of possible respiratory frequencies and amplifying nasal airflow. In such condition, we observed that LFP frequency could reflect respiratory frequency during SWS and REM states, whereas it was not observable in ambient air condition. We concluded that the global brain respiratory drive was determined by respiratory regime and not by the brain state itself. We chose CO_2_ over respiratory nuclei stimulation^35^ to modify respiratory regime mainly because the method was easier to implement and not invasive. However, CO_2_ use remains questionable because of its known effect on locus coeruleus^36, 37^, on vegetative functions (increase in heart and respiratory rates), and on other complex behaviors such as anxiety^38^. Nevertheless, the very low concentration used here (5%) did not cause any significant change in the animals’ behavior. Once habituated to CO_2_ exposure, animals slept normally.

An interesting point in our study is our demonstration of an “optimal” respiratory regime for brain entrainment in rats, consisting in a deep (around 20 mL/sec) and long (around 0.15 s) inspiration. Indeed, a respiratory cycle based analysis allowed us to study LFP-respiration coupling in details. We observed that a deep inspiration alone was not sufficient to entrain non-olfactory structures: if it was the case, we should have observed a large respiratory drive during active exploration where flow rates are the highest (see Fig. 2), with inspiration being very ample but also very short. Similarly, a long inspiration alone was not sufficient to drive non-olfactory areas since SWS and REM states exhibited the longest inspirations while entrainment was restricted to olfactory areas and PFC. During sleep states, inspiration was very long but also very low in amplitude. Finally, the only state presenting an optimal breathing regime, with a deep and long inspiration, is the quiet waking state. We previously showed in the anesthetized tracheotomized rat that the respiratory drive in the OB depended on a trade-off between frequency and flow rate in the nasal cavity^30, 32^. The present study extends this finding to non-olfactory areas and more importantly to the ecological condition of freely moving animal. Because brain respiratory-drive disappears when nasal respiration is by-passed or when OB activity is suppressed^10, 12, 15, 19, 20^, it is tempting to conclude from our results that the duration and flow rate of the airflows circulating in the nasal cavity, mechanically stimulating the olfactory epithelium^22^, and thus activating the OB, determines the respiratory drive in the brain. In such view, the OB would represent the main driving force of the respiration influence onto the brain. However, given that higher airflows implies a deeper breathing and thus a different recruitment of respiratory centers and motor control, the effect of other pathways cannot be excluded.

We observed that AE state was peculiar in that sense that non-olfactory structures (except PFC) exhibited a kind of resistance to respiratory coupling in the high range of respiration frequency (> 5 Hz), even in CO_2_ condition. We can formulate different hypotheses to try to explain this phenomenon. First, CO_2_ use, while resulted in inspiration depth increase, did not modify inspiration duration during AE (see Supplementary Fig. S2). Since, as described also by others^17^, more posterior areas are less susceptible to respiration drive, we could assume that these structures would require a longer inspiration duration to be respiration-entrained. Second, AE state is dominated by sniffing epochs where a strong hippocampal theta rhythm coupling has been described^17^. It could be possible that a strong theta coupling preclude respiratory drive in structures where the two rhythms compete. During such episodes, hippocampal theta and respiration frequencies completely overlap (see spectra in Fig. 3) so that it was not possible to disentangle theta-coupling from respiration-coupling in this frequency range^17^. We have also to keep in mind that sniffing act is likely a very different mechanism than a high rate respiration, which involves other brain structures, maybe explaining that non-olfactory brains structures could be less responsive to nasal airflow during this behavior.

Our study pointed out that all brain structures were not respiratory-coupled to the same extent. This has been previously reported that, in either REM or exploration, respiratory drive was most prominent in frontal regions (OB, prelimbic cortex, anterior cingulate cortex)^17, 33^. Interestingly, PFC seems particularly sensitive to respiratory drive. Indeed, it is the only non-olfactory structure that appeared respiration-coupled during the four brain states, whatever the respiratory regime. Individual data of LFP-respiration coupling index as a function of inhalation amplitude or duration (Fig. 7) emphasized the strong and systematic coupling of this structure with respiration. PFC has been previously shown to be very well tuned to the 4 Hz respiratory frequency during animal freezing^39–42^. Here, the strong tuning of PFC with respiratory rhythm holds whatever the respiratory frequency as previously evoked^42^. This suggests that PFC could function as a hub structure, able to tune its activity to different areas according to the respiration frequency. Yet, PFC activity is coherent with basolateral amygdala during freezing when the animal breathes in the 2-4 Hz range^39^, but it can also synchronize with hippocampal activity in the 7–8 Hz range^43–45^.

In their review, Heck et al.^46^ emphasized the potential role of respiration in organizing cortical activity in memory processes, notably by its ability to phase-lock hippocampal sharp wave ripples during wakening^15^. Our demonstration that brain structures are collectively and strongly susceptible to respiratory drive during quiet waking suggests that memory processes could be favored during this particular rest state. Our further demonstration that nasal airflow dynamics are crucial in the extent of brain entrainment by respiration could explain why memory consolidation is improved by nasal versus oral breathing^6^. The question remains about testing if an equivalent of the quiet state exists in human. We assume that such a state, characterized by a generalized brain entrainment on respiration, could be reached during deep-breathing practice.

The crucial role of the olfactory pathway in brain respiratory-drive is nowadays largely recognized, and particularly well emphasized in review papers^2, 46–49^. But credit where credit is due: in their excellent opinion paper, Fontanini and Bower^50^ were the first in 2006 to clearly posit that “[…] it might be that the pan-cortical correlations that particularly characterize slow-wave sleep and meditation actually depend on an afferent input signal generated within the olfactory system.” Our work is definitely in line with this thinking and that of a “sniffing brain” they developed. Moreover, our new data answers in the affirmative to the following question^46^: “Does the change in sniff frequency (for rats, rabbits, cats, and mice) or duration (for humans) alter the cortical circuit by driving the OB differently via the olfactory nerve […]”. Finally, our results suggest that nasal airflow adjustments by variation in respiratory regime could be an effective tool for influencing brain activity. Notably, the slow and deep breathing associated to rest state seems to be an optimal regime to achieve a respiratory drive onto the whole brain.

## Methods

### Experimental procedures

#### Animal care

Thirteen male Long Evans rats (Janvier Laboratories, Le Genest Saint Isle, France; 8 weeks old, ~ 250 to 300 g at the start of the experiment) were used and housed in groups of four in a temperature (22 ± 1 ◦C) and humidity (55 ± 10%) controlled room and exposed to a 12/12 h light/dark cycle (light onset, 6:00 am). Experiments were conducted during the light period (between 9:00 am and 3:00 pm). Food and water were available ad libitum. All experiments were carried out in accordance with Directive 2010/63/EU of the European Parliament and of the Council of the European Union regarding the protection of animals used for scientific purposes and in compliance with the ARRIVE guidelines. The experimental protocols were approved by the National Ethics Committee “Animal Experimentation Committee of Univ. Claude Bernard Lyon 1—CEEA-55” (Agreement APAFIS #17088).

#### Animal preparation

Preparation consisted in electrode implantations in different brain areas. Anesthesia was induced by an initial dose of a mixture of chloral hydrate and sodium pentobarbital (intraperitoneal; Equithesin 3 ml/kg) and maintained with additional doses as needed. Rats were administered with anti-inflammatory and analgesic treatment (subcutaneous; carprofen 2 mg/kg or meloxicam 0.2 mg/kg) immediately after surgery and during several postoperative days if necessary. After a minimal 10 days’ period of recovery, animals were implanted with monopolar stainless steel LFP recording electrodes (diameter: 100 µm, 200–800 kΩ impedance, California Fine Wire) soldered to a copper wire (diameter: 250 µm). Electrodes were positioned stereotactically into the left cortical hemisphere in 6 (n = 8 rats) or 7 (n = 5 rats) brain areas (see Table 1 for coordinates and sample size) comprising the olfactory bulb (OB), anterior piriform cortex (AP), dorsal part of the hippocampus CA1 (CA1) and dentate gyrus (DG), primary visual cortex (V1), primary somatosensory barrel cortex (S1), and prefrontal cortex (PFC). Electrophysiological activity was recorded while the electrode was lowered. Positioning of the recording electrode in the AP was determined by the shape of evoked field potential induced by stimulation of a bipolar OB electrode placed close to the mitral cell layer. The final location was selected to where the evoked potentials had the largest amplitude (before reversal). The stimulation electrode was then replaced by a monopolar recording electrode in the OB. The depth of the recording electrodes in the OB and CA1 was adjusted using their large multiunit activity, to the level of the mitral cell layer and just below the pyramidal layer, respectively. Positioning of the other recording electrodes tips (DG, S1, V1, PFC) was achieved stereotactically. Each electrode was individually fixed to the skull using dental cement. A reference wire was connected to a skull golden screw located above the posterior portion of the contralateral cortical hemisphere. Two anchor screws were also inserted in the contralateral side to secure the implant. Each electrode was attached to a 32-pin electrode interface board (EIB, NeuraLynx, Inc, USA, ViewPoint France) combined with an omnetics connector and centered on the animal’s head.

#### LFP and respiration recording

Signals were acquired by telemetry using a 32 channels wireless recording system (W32 headstage, TBSI, ViewPoint France). Signals were sampled at 15 kHz, amplified (gain 800×) and recorded via an acquisition card (USB-2533, Measurement Computing, Norton, MA). Signals were acquired using custom-made open-source software (pyacq, https://github.com/pyacq/pyacq) and stored on a computer for offline analysis.

Respiratory activity was recorded by using whole-body plethysmography. Plethysmograph (EMKA Technologies, France) was previously described in detail^51, 52^.

One camera (B/W CMOS PINHOLE camera) was placed in a corner of the cage in order to monitor the animal behavior.

#### Recording sessions

Before starting recording sessions, animals were habituated to the plethysmography cage during 2 sessions of 20 min. At the end of this period, animals were completely familiarized with the environment and did not show any sign of stress.

##### Ambient air

Recording sessions then started. Six sessions (one session per day) of 2 h under ambient air were recorded for each animal. During the first minutes, animals alternated between active exploration and quiet rest. After a certain amount of time, variable between animals (10–30 min), rats started to sleep, with SWS and REM states alternation.

##### CO_2_-enriched air

In some experiments, we used a 5% CO_2_-enriched air in the plethysmograph to change respiration regime. The choice of 5% concentration was based on respiratory physiology experiments, where the CO_2_ concentration usually used is 8%^53^. It has been shown that a concentration of 10% can increase immobility bouts in Long-Evans rats, suggesting a certain level of anxiety^54^. We therefore searched for the lowest concentration able to induce a change in the amplitude and/or respiratory frequency. Our criteria were that: (1) animal could fall asleep with the same delay as under ambient air, (2) no signs of stress (no increase in episodes of immobility or grooming) were shown, (3) sniffing frequency returned to baseline in less than 5 min after CO_2_ stopping in the plethysmograph. Pilot experiments (n = 4, not shown) allowed us to define 5% CO_2_ concentration as meeting all these criteria. Following the three ambient air recording sessions, five animals were recorded during 3 new sessions under CO_2_-enriched air (one session per day). To diffuse CO_2_ enriched air, a 10% CO_2_—air container (Air Liquide, France) was connected to the plethysmograph. Pure air was mixed with CO_2_ (50/50) so that the final concentration was 5%. Under CO_2_, the recording sessions were limited to 2 h. Exposure of animals three times 2 h was not sufficient to induce chronic hypercapnia^55^.

### Data analysis

#### Respiratory signal

Respiratory signals were acquired and extracted using previously described method^51^ allowing accurate measurements of different respiratory parameters in behaving animals. The detection of the respiratory cycles was achieved using an enhanced version of the algorithm described in a previous study^56^. This algorithm performs two main operations: signal smoothing for noise reduction, and detection of zero-crossing points to define accurately the inspiration and expiration phase starting points. For each respiratory cycle, inspiration and expiration peak flow rate (iPF, ePF), inspiration duration and expiration duration (iDur, eDur) were measured. Instantaneous respiratory frequency (Freq) was determined as the inverse of the respiratory cycle (inspiration plus expiration) duration.

### States scoring

Based on video, electrophysiological, and breathing recordings, two well-trained experimenters visually coded four vigilance states by inspection of behaviors, respiration, and associated LFP spectral features (see Fig. 1). State was classified as active exploration (AE) if the animal engaged in exploratory behavior (locomotion, whisking, and sniffing), with a high amplitude and > 5 Hz respiration^33^ during at least three cycles, and a cortical LFPs expressing high gamma (30–55 Hz) power density. In quiet waking (QW), the animal was immobile (standing or sitting quietly) with a high amplitude and < 5 Hz respiration, cortical LFPs expressed relatively high gamma activity. In slow wave sleep (SWS), the animal was lying immobile with eyes closed and slow and extremely regular respiratory movements. LFPs were characterized by prominent delta waves (1–4 Hz). In rapid eye movements sleep (REM), the animal was immobile with eyes closed. Breathing was irregular, with epochs of low and slow amplitude alternating with short epochs of higher frequency and amplitude (see Figs. 1 and 2). LFPs were low in amplitude and expressed very high theta and gamma power. Intermediary states, where LFP features were not very clear, were not coded.

### Electrophysiological signals

Data were analyzed using custom-written scripts with the python language and its scientific ecosystem tool suite.

#### Spectral analysis

The power spectral density (PSD) of the LFP signals was calculated using the continuous Morlet wavelet transform^57^ instead of the classical windowed Fourier transform (FFT). The Morlet wavelet estimated the amplitude of the signal across time and frequency. The obtained time–frequency map was then segmented in periods of interest with variable durations and averaged on the time axis. We made this choice mainly because low frequency analyses require very long windows with FFT methods while the Morlet wavelet transform is a continuous representation along time. Segmenting state periods with varying durations combined with fixed windowed method would be sub-optimal whereas the continuous representation in the Morlet time frequency map makes the process easier and more accurate. The drawback with our approach is that the final PSD is smoother on the frequency domain than a standard FFT. To compare spectra shape across channels and states while removing some variability across rats, we normalized all spectra from the same rat and channel to an average area of 1 across states. We thus obtained spectra whose amplitude was similar across rats and channels, and was still quantitatively comparable across states.

#### Coherence analysis

For the computation, the signal was down sampled to 1000 Hz. LFP-respiration coherence was calculated with a custom-modified version of Scipy coherence function. Indeed, since coherence is based on FFT window method then the outcome is biased by the total duration of the period. In our case, to compensate the great difference in duration between states, we randomly sub-sampled an equal number of non-juxtaposed epochs of each state and then used the classic coherence with no overlap on these epochs (256 epochs of 6 s). Coherence between LFP and respiration was compared against chance using a surrogate-based statistical testing. Surrogate were obtained by preserving the LFP and respiration spectra using a four-steps process: (1) shuffling (n = 100) of 256 epochs, (2) coherence computing, (3) extraction of the maximum coherence value, and (4) computation of the distribution of surrogate values. Our first statistical analysis of the coherence at the population level compared the distribution of coherence maximal values to the average of surrogate spectrum values measured at the same frequency (computed independently for each rat). The comparison across rats was then done using a linear mixed-effect model including also the animal state and the recorded structure as fixed effects, and all their interactions. To get a convergent model, only the intercept and the animal state were used as random effects but we checked that the full model (with all random effects) gave similar results. Besides, coherence values were log-transformed in order to insure homoscedasticity of residuals. Normality of residuals was checked after model fitting. ANOVA on fixed effects was obtained by computing degree of freedom with the Kenward-Roger approximation. Finally, post-hoc tests on fitted model used standard paired t-test (corrected for multiple comparisons with the false discovery rate method). Model fitting and post-hoc tests were done with software R 3.4.4 and libraries afex 0.25-1, lme4 1.1-21 and emmeans 1.4.2.

The same linear mixed model fitting approach and statistical analysis was used in a second statistical analysis in order to compare the distributions of coherence maximal values in air and CO_2_ conditions.

#### Covariation maps and coupling index

To study frequency-frequency coupling between LFP signals and respiration, we designed a homemade method that allowed tracking instantaneous frequency synchrony^42^. To do so, for each detected respiratory cycle, the frequency was estimated as 1/cycle duration and the time course of the instantaneous respiratory frequency was extracted. In parallel, the continuous Morlet scalogram for the LFP signal was computed in our frequency band of interest (0.8–10 Hz). In each time bin (4 ms), we extracted the frequency of the point of maximum instantaneous power. Finally, this sequence of frequencies gave us the time course of the LFP predominant instantaneous frequency. From the two times series obtained (instantaneous respiration frequency and predominant instantaneous LFP frequency), a 2D matrix histogram was built, with the respiratory frequency represented on the x-axis and the LFP frequency on y-axis (2-dimensional distribution of joined LFP-respiration frequencies). This 2D histogram, called covariation map, was normalized so that the total sum equaled 1, and point density was represented on a color scale ranging from blue to yellow as the density of points increased. A coupling between respiration frequency and LFP frequency can be assumed when a high density (i.e., yellow color) was observed along the diagonal of the covariation map (see Fig. 5 for an illustration). Conversely, a non-correlated Gaussian shape indicated that the frequency of respiration and the main frequency of LFP varied independently. The two possibilities can co-occur on the same covariation map.

Based on this map, we were then able to introduce a new metric: the sum of density along the diagonal. To assess the density of the diagonal, we applied a smoothing Gaussian kernel ($\sigma$ = 0.58 Hz) to the covariation maps before summing the diagonal. This metric estimated the coupling strength between respiration and LFP with a factor in 0–1 range where 1 indicates that respiration frequency is always the dominant LFP frequency and 0 signals the two frequencies are never equal. We named “coupling index” this custom metric. To validate the accuracy of this metric, we constructed surrogates (n = 100) by applying random time-shift (with the same method we used in “Coherence analysis”, see above) between the respiration time series and LFP time series. This procedure allows to remove the instantaneous coupling but maintains the global frequency distribution of both time series.

Statistical analysis of coupling index at the population level was performed using linear mixed models similarly as described for the coherence (see above). However to get homoscedasticity of residuals, the coupling index (CI), was transformed with log(− log(CI)) when comparing air and shuffle data, and not transformed when comparing air and CO_2_ data.

The two time-series (respiration frequency and LFP frequency) describing the covariation map could be segmented in short periods of variable duration before computing the covariation map. This segmentation allowed to group respiratory cycles with common characteristic, for instance iPF or iDur. For each of these groups of cycles, we could compute a covariation map and the corresponding coupling index. Ultimately, we were able to display a coupling index vs “inspiration peak flow rate” (or duration) plot across animal state and on an animal-by-animal basis. In some cases, a total duration of 60 s was not reached: the missing values were then not considered for the computation.

While looking at latter analyses, we often noticed the existence of a local maximum of coupling index for intermediate values of a given respiratory cycle characteristic. In order to statistically assess the existence of this intermediate maximum, we fitted our data with generalized linear mixed-effect models. Model fixed-effects included a first or second order polynomial of the respiratory cycle characteristic, while random effects always included a second order polynomial. Data were grouped per animal (distinct models have been done for each area). The link function was a logarithm and the variance described with a Gamma function (family function). We visually checked homoscedasticity and normality of the residuals after model fitting. Comparison between nested models was obtained with a Wald chi-square test. Mixed-model analysis was done with software R 3.4.4 and library lme4 1.1-21.

## Supplementary Information

Supplementary Information.

## Data Availability

All data and scripts at any stage of analysis are available upon reasonable requests from Nathalie Buonviso (nathalie.buonviso@cnrs.fr).

## References

[1] Fries P (2005). A mechanism for cognitive dynamics: Neuronal communication through neuronal coherence. Trends Cogn. Sci..

[2] Heck DH (2017). Breathing as a fundamental rhythm of brain function. Front. Neural Circuits.

[3] Tort ABL, Brankačk J, Draguhn A (2018). Respiration-entrained brain rhythms are global but often overlooked. Trends Neurosci..

[4] Hegoburu C (2011). The RUB cage: Respiration-ultrasonic vocalizations-behavior acquisition setup for assessing emotional memory in rats. Front. Behav. Neurosci..

[5] Kurnikova A, Moore JD, Liao S-M, Deschênes M, Kleinfeld D (2017). Coordination of orofacial motor actions into exploratory behavior by rat. Curr. Biol..

[6] Arshamian A, Iravani B, Majid A, Lundström JN (2018). Respiration modulates olfactory memory consolidation in humans. J. Neurosci..

[7] Nakamura NH, Fukunaga M, Oku Y (2018). Respiratory modulation of cognitive performance during the retrieval process. PLoS ONE.

[8] Iwabe T, Ozaki I, Hashizume A (2014). The respiratory cycle modulates brain potentials, sympathetic activity, and subjective pain sensation induced by noxious stimulation. Neurosci. Res..

[9] Li S, Laskin JJ (2006). Influences of ventilation on maximal isometric force of the finger flexors. Muscle Nerve.

[10] Zelano C (2016). Nasal respiration entrains human limbic oscillations and modulates cognitive function. J. Neurosci..

[11] Perl O (2019). Human non-olfactory cognition phase-locked with inhalation. Nat. Hum. Behav..

[12] Ito J (2014). Whisker barrel cortex delta oscillations and gamma power in the awake mouse are linked to respiration. Nat. Commun..

[13] Nguyen Chi, V. *et al.* Hippocampal respiration-driven rhythm distinct from theta oscillations in awake mice. *J. Neurosci.* **36**, 162–177 (2016). 10.1523/JNEUROSCI.2848-15.2016 PMC6601786 26740658

[14] Biskamp J, Bartos M, Sauer J-F (2017). Organization of prefrontal network activity by respiration-related oscillations. Sci. Rep..

[15] Liu Y, McAfee SS, Heck DH (2017). Hippocampal sharp-wave ripples in awake mice are entrained by respiration. Sci. Rep..

[16] Zhong W (2017). Selective entrainment of gamma subbands by different slow network oscillations. Proc. Natl. Acad. Sci..

[17] Tort ABL (2018). Parallel detection of theta and respiration-coupled oscillations throughout the mouse brain. Sci. Rep..

[18] Herrero JL, Khuvis S, Yeagle E, Cerf M, Mehta AD (2018). Breathing above the brain stem: Volitional control and attentional modulation in humans. J. Neurophysiol..

[19] Yanovsky Y, Ciatipis M, Draguhn A, Tort ABL, Brankačk J (2014). Slow oscillations in the mouse hippocampus entrained by nasal respiration. J. Neurosci..

[20] Lockmann ALV, Laplagne DA, Leão RN, Tort ABL (2016). A Respiration-coupled rhythm in the rat hippocampus independent of theta and slow oscillations. J. Neurosci..

[21] Ravel N, Pager J (1990). Respiratory patterning of the rat olfactory bulb unit activity: Nasal versus tracheal breathing. Neurosci. Lett..

[22] Grosmaitre X, Santarelli LC, Tan J, Luo M, Ma M (2007). Dual functions of mammalian olfactory sensory neurons as odor detectors and mechanical sensors. Nat. Neurosci..

[23] Cenier T (2008). Odor vapor pressure and quality modulate local field potential oscillatory patterns in the olfactory bulb of the anesthetized rat. Eur. J. Neurosci..

[24] Chaput MA, Buonviso N, Berthommier F (1992). Temporal patterns in spontaneous and odour-evoked mitral cell discharges recorded in anaesthetized freely breathing animals. Eur. J. Neurosci..

[25] Buonviso N (2003). Rhythm sequence through the olfactory bulb layers during the time window of a respiratory cycle. Eur. J. Neurosci..

[26] Cenier T (2009). Respiration-gated formation of gamma and beta neural assemblies in the mammalian olfactory bulb. Eur. J. Neurosci..

[27] Briffaud V, Fourcaud-Trocmé N, Messaoudi B, Buonviso N, Amat C (2012). The relationship between respiration-related membrane potential slow oscillations and discharge patterns in mitral/tufted cells: What are the rules?. PLoS ONE.

[28] Fourcaud-Trocmé N, Briffaud V, Thévenet M, Buonviso N, Amat C (2018). In vivo beta and gamma subthreshold oscillations in rat mitral cells: Origin and gating by respiratory dynamics. J. Neurophysiol..

[29] Oka Y, Takai Y, Touhara K (2009). Nasal airflow rate affects the sensitivity and pattern of glomerular odorant responses in the mouse olfactory bulb. J. Neurosci..

[30] Courtiol E (2011). Individual and synergistic effects of sniffing frequency and flow rate on olfactory bulb activity. J. Neurophysiol..

[31] Courtiol E (2011). Reshaping of bulbar odor response by nasal flow rate in the rat. PLoS ONE.

[32] Esclassan F (2012). Faster, deeper, better: The impact of sniffing modulation on bulbar olfactory processing. PLoS ONE.

[33] Rojas-Líbano, D., Wimmer del Solar, J., Aguilar-Rivera, M., Montefusco-Siegmund, R. & Maldonado, P. E. Local cortical activity of distant brain areas can phase-lock to the olfactory bulb’s respiratory rhythm in the freely behaving rat. *J. Neurophysiol.* **120**, 960–972 (2018). 10.1152/jn.00088.2018 29766764

[34] Cavelli M (2020). Nasal respiration entrains neocortical long-range gamma coherence during wakefulness. Eur. J. Neurosci..

[35] Burke PGR (2014). Optogenetic stimulation of adrenergic C1 neurons causes sleep state-dependent cardiorespiratory stimulation and arousal with sighs in rats. Am. J. Respir. Crit. Care Med..

[36] Oyamada Y, Ballantyne D, Mückenhoff K, Scheid P (1998). Respiration-modulated membrane potential and chemosensitivity of locus coeruleus neurones in the in vitro brainstem-spinal cord of the neonatal rat. J. Physiol..

[37] Pineda J, Aghajanian GK (1997). Carbon dioxide regulates the tonic activity of locus coeruleus neurons by modulating a proton- and polyamine-sensitive inward rectifier potassium current. Neuroscience.

[38] Oikawa S, Hirakawa H, Kusakabe T, Nakashima Y, Hayashida Y (2005). Autonomic cardiovascular responses to hypercapnia in conscious rats: the roles of the chemo- and baroreceptors. Auton Neurosci..

[39] Dejean C (2016). Prefrontal neuronal assemblies temporally control fear behaviour. Nature.

[40] Karalis N (2016). 4-Hz oscillations synchronize prefrontal-amygdala circuits during fear behavior. Nat. Neurosci..

[41] Moberly AH (2018). Olfactory inputs modulate respiration-related rhythmic activity in the prefrontal cortex and freezing behavior. Nat. Commun..

[42] Dupin, M., Garcia, S., Boulanger-Bertolus, J., Buonviso, N. & Mouly, A.-M. New insights from 22-kHz ultrasonic vocalizations to characterize fear responses: Relationship with respiration and brain oscillatory dynamics. *eNeuro* **6** (2019). 10.1523/ENEURO.0065-19.2019 PMC6506822 31064837

[43] Jones MW, Wilson MA (2005). Theta rhythms coordinate hippocampal-prefrontal interactions in a spatial memory task. PLoS Biol..

[44] Siapas AG, Lubenov EV, Wilson MA (2005). Prefrontal phase locking to hippocampal theta oscillations. Neuron.

[45] Colgin LL (2011). Oscillations and hippocampal-prefrontal synchrony. Curr. Opin. Neurobiol..

[46] Heck DH, Kozma R, Kay LM (2019). The rhythm of memory: How breathing shapes memory function. J. Neurophysiol..

[47] Zaccaro, A. *et al.* How breath-control can change your life: A systematic review on psycho-physiological correlates of slow breathing. *Front. Hum. Neurosci.* **12** (2018). 10.3389/fnhum.2018.00353 PMC6137615 30245619

[48] Kontaris I, East BS, Wilson DA (2020). Behavioral and neurobiological convergence of odor, mood and emotion: A review. Front. Behav. Neurosci..

[49] Maric V, Ramanathan D, Mishra J (2020). Respiratory regulation & interactions with neuro-cognitive circuitry. Neurosci. Biobehav. Rev..

[50] Fontanini A, Bower JM (2006). Slow-waves in the olfactory system: An olfactory perspective on cortical rhythms. Trends Neurosci..

[51] Courtiol E (2014). Sniff adjustment in an odor discrimination task in the rat: Analytical or synthetic strategy?. Front. Behav. Neurosci..

[52] Lefèvre L (2016). Significance of sniffing pattern during the acquisition of an olfactory discrimination task. Behav. Brain Res..

[53] Ramanantsoa N (2011). Breathing without CO_2_ chemosensitivity in conditional Phox2b mutants. J. Neurosci..

[54] Winter A, Ahlbrand R, Naik D, Sah R (2017). Differential behavioral sensitivity to carbon dioxide (CO_2_) inhalation in rats. Neuroscience.

[55] Reichart E, Cogniel O, Brothier G, Marchand M, Colas T (1972). Blood acid-base status in the rat during 2 months of exposure to hypercapnia. Pflugers Arch..

[56] Roux SG (2006). Respiratory cycle as time basis: An improved method for averaging olfactory neural events. J. Neurosci. Methods.

[57] Kronland-Martinet R, Morlet J, Grossmann A (1987). Analysis of sound patterns through wavelet transforms. Int. J. Pattern Recogn. Artif. Intell..

