# Supplementary material for: The deep and slow breathing characterizing rest favors brain respiratory-drive
Source: Sci Rep. 2021 Mar 29;11:7044. doi: 10.1038/s41598-021-86525-3 (PMC8007577; doi:10.1038/s41598-021-86525-3)

**The deep and slow breathing characterizing rest favors brain respiratory-drive**

**Baptiste Girin^1,3^, Maxime Juventin^1,3^, Samuel Garcia^1^, Laura Lefèvre^2^, Corine Amat^1^, Nicolas Fourcaud-Trocmé^1,4^, Nathalie Buonviso^1,4*^**

1 Lyon Neuroscience Research Center (CRNL), Inserm U 1028, CNRS UMR 5292, University Lyon 1, Bron, 69675, France.

2 Present address: Medical Research Council Brain Network Dynamics Unit, Nuffield Department of Clinical Neurosciences, University of Oxford, OX1 3TH, Oxford, UK.

3 These authors contributed equally

4 Last co-authors

*corresponding author: [nathalie.buonviso@cnrs.fr](mailto:nathalie.buonviso@cnrs.fr)

**Supplementary material**

Supplementary Table S1: LFP-respiration coherence. For each state (AE, QW, SWS, and REM) and each structure (OB, AP, PFC, S1, CA1, V1, DG), actual and surrogates maximum coherence values between LFP and respiration were compared using the process described in Methods. ANOVA analysis of fixed effects showed significant interactions of data type (actual or surrogate data) and both state (F_3, 375.7_=12.4, p < 0.0001) and structure (F_6, 375.7_=2.94, p < 0.0001). We thus checked statistical difference between actual and surrogate data across rats for each state/structure pairs. Results are in the table below. Freq max is the average frequency (in Hz) across animals at the maximum coherence. df = 375.68 for all the lines.

| **state** | **structure** | **freq max** | **actual** | **surrogates** | **t.ratio** | **p.value** |
| --- | --- | --- | --- | --- | --- | --- |
| **AE** | **OB** | 7.571 | 0.49 | 0.0041 | 20.04 | p<0.001 |
|  | **AP** | 5.606 | 0.28 | 0.0037 | 23.63 | p<0.001 |
|  | **PFC** | 5.333 | 0.40 | 0.0040 | 16.95 | p<0.001 |
|  | **CA1** | 2.727 | 0.12 | 0.0037 | 18.53 | p<0.001 |
|  | **DG** | 2.800 | 0.09 | 0.0039 | 15.74 | p<0.001 |
|  | **S1** | 2.167 | 0.14 | 0.0037 | 17.63 | p<0.001 |
|  | **V1** | 4.250 | 0.09 | 0.0040 | 14.11 | p<0.001 |
| **QW** | **OB** | 3.479 | 0.43 | 0.0039 | 20.95 | p<0.001 |
|  | **AP** | 2.181 | 0.37 | 0.0040 | 24.70 | p<0.001 |
|  | **PFC** | 2.100 | 0.49 | 0.0040 | 17.47 | p<0.001 |
|  | **CA1** | 1.833 | 0.21 | 0.0041 | 21.94 | p<0.001 |
|  | **DG** | 1.848 | 0.15 | 0.0035 | 20.17 | p<0.001 |
|  | **S1** | 1.894 | 0.18 | 0.0039 | 20.02 | p<0.001 |
|  | **V1** | 1.944 | 0.13 | 0.0040 | 16.88 | p<0.001 |
| **SWS** | **OB** | 1.619 | 0.07 | 0.0039 | 12.01 | p<0.001 |
|  | **AP** | 1.788 | 0.06 | 0.0040 | 12.94 | p<0.001 |
|  | **PFC** | 1.067 | 0.05 | 0.0042 | 7.80 | p<0.001 |
|  | **CA1** | 3.106 | 0.03 | 0.0040 | 10.73 | p<0.001 |
|  | **DG** | 2.061 | 0.03 | 0.0038 | 10.40 | p<0.001 |
|  | **S1** | 1.533 | 0.04 | 0.0038 | 12.48 | p<0.001 |
|  | **V1** | 3.685 | 0.04 | 0.0040 | 9.80 | p<0.001 |
| **REM** | **OB** | 2.533 | 0.11 | 0.0038 | 11.47 | p<0.001 |
|  | **AP** | 1.139 | 0.11 | 0.0039 | 13.25 | p<0.001 |
|  | **PFC** | 1.111 | 0.12 | 0.0034 | 8.92 | p<0.001 |
|  | **CA1** | 3.889 | 0.06 | 0.0040 | 10.05 | p<0.001 |
|  | **DG** | 1.167 | 0.07 | 0.0040 | 11.57 | p<0.001 |
|  | **S1** | 2.333 | 0.07 | 0.0038 | 10.21 | p<0.001 |
|  | **V1** | 1.167 | 0.06 | 0.0040 | 10.09 | p<0.001 |

Supplementary Figure S1: **A**: Surrogate covariation maps, averaged across rats, obtained from shuffling LFP signals recorded under ambient air. Y-axis represents LFP frequency and X-axis respiratory frequency. We can observe that diagonal are not highlighted in surrogate data. Note there are 100 shuffled maps for each rat, averaged per rat and then across rat for the figure. **B**: Coupling Index comparison between actual air (act.) and surrogate (surr.) conditions. For each animal, 100 shuffled maps were computed allowing computing the distribution of shuffled maps coupling index, but standard deviations are smaller than point size on the figure.


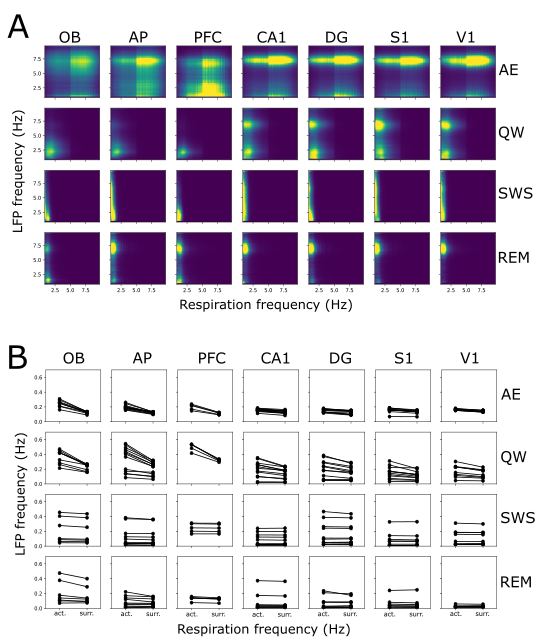


Supplementary Table S2: Coupling Index between LFP main frequency and respiration frequency. For each state (AE, QW, SWS, and REM) and each structure (OB, AP, PFC, S1, CA1, V1, DG), actual and surrogates Coupling Index between LFP and respiration were compared using the process described in Methods. ANOVA analysis of fixed effects showed significant two-way interactions of data type (actual or surrogate data) with both animal state (F_3.448.3_=12.4, p < 0.0001) and structure (F_6.448.3_=2.94, p = 0.0008). We thus checked statistical difference between actual and surrogate data across rats for each state/structure pairs. Results are in the table below. df = 448.31 for all the lines.

However, because ’Structure’ was not included in model random slopes, we also ran the same model excluding olfactory structures (OB, AP) and PFC in order to highlight smaller effects in non-olfactory structures. It showed a significant two-way interaction between data type and animal state (F_3, 272.5_=3.10, p = 0.027) but not data type and channel (F_3, 272.5_=0.02, p = 0.97). Post-hoc tests showed a significant effect of data type in QW only (T tests, df = 273; QW: t=-3.47, p = 0.002; AE: t = -2.14, p = 0.10; SWS: t = -0.032, p = 1; REM: t = 0.33, p = 1) emphasizing that QW is the state with the most significant coupling whatever the structure.

| **State** | **Structure** | **Actual** | **Surrogate** | **t ratio** | **p value** |
| --- | --- | --- | --- | --- | --- |
| **AE** | **OB** | 0.25 | 0.12 | -4.24 | 0.00 |
|  | **AP** | 0.20 | 0.12 | -3.37 | 0.00 |
|  | **PFC** | 0.20 | 0.11 | -2.48 | 0.06 |
|  | **CA1** | 0.16 | 0.13 | -1.27 | 0.52 |
|  | **DG** | 0.15 | 0.13 | -1.04 | 0.63 |
|  | **S1** | 0.16 | 0.13 | -1.08 | 0.63 |
|  | **V1** | 0.16 | 0.14 | -0.88 | 0.71 |
| **QW** | **OB** | 0.36 | 0.23 | -4.11 | 0.00 |
|  | **AP** | 0.34 | 0.21 | -4.64 | 0.00 |
|  | **PFC** | 0.50 | 0.32 | -4.03 | 0.00 |
|  | **CA1** | 0.19 | 0.14 | -2.09 | 0.15 |
|  | **DG** | 0.20 | 0.16 | -1.76 | 0.26 |
|  | **S1** | 0.15 | 0.11 | -1.74 | 0.26 |
|  | **V1** | 0.17 | 0.13 | -1.35 | 0.50 |
| **SWS** | **OB** | 0.21 | 0.19 | -0.38 | 1.00 |
|  | **AP** | 0.12 | 0.12 | -0.35 | 1.00 |
|  | **PFC** | 0.25 | 0.24 | -0.07 | 1.00 |
|  | **CA1** | 0.08 | 0.08 | 0.01 | 1.00 |
|  | **DG** | 0.16 | 0.15 | -0.11 | 1.00 |
|  | **S1** | 0.07 | 0.07 | 0.03 | 1.00 |
|  | **V1** | 0.11 | 0.11 | 0.00 | 1.00 |
| **REM** | **OB** | 0.21 | 0.17 | -1.01 | 0.63 |
|  | **AP** | 0.08 | 0.07 | -0.18 | 1.00 |
|  | **PFC** | 0.13 | 0.12 | -0.39 | 1.00 |
|  | **CA1** | 0.07 | 0.06 | 0.12 | 1.00 |
|  | **DG** | 0.06 | 0.06 | 0.26 | 1.00 |
|  | **S1** | 0.05 | 0.05 | 0.20 | 1.00 |
|  | **V1** | 0.02 | 0.02 | 0.09 | 1.00 |


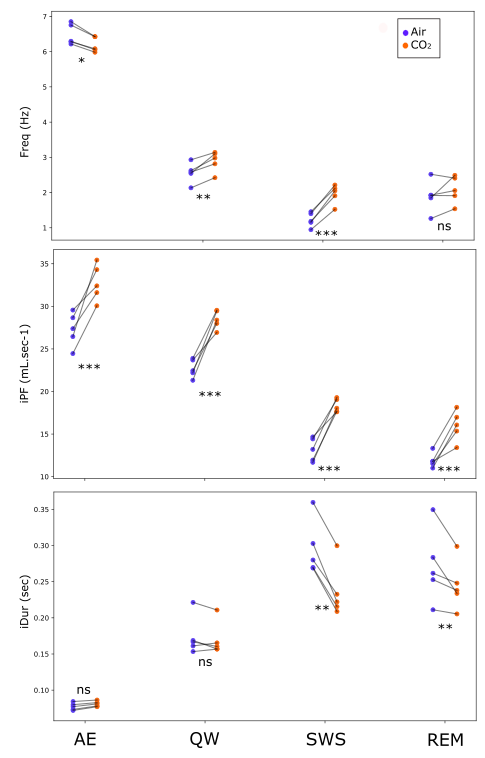
Supplementary Figure S2: Difference of respiration frequency (Freq. **top panel**), inspiration duration (iDur, **middle panel**) and inspiration peak flowrate (iPF, **bottom panel**) between ambient AIR and CO_2_-enriched conditions. One data point per rat/condition/state. See statistics in Table below. Repeated measure ANOVA with condition and state as within factors showed significant effect of the state-condition interaction for Freq and iDur (Freq: *F*_3.12_ = 26.7, p < 0.001; iDur: *F*_3.12_ = 22.6, p < 0.001) and a significant effect of condition alone for iPF ( *F*_3.12_ = 133.3, p < 0.001). Table: Each parameters of each state (AE active exploration. QW quiet waking. SWS slow-wave sleep. REM rapid eye movements sleep) is compared between ambient AIR and CO_2_- enriched air using paired T-test with Holm correction. N = 5 rats, * p < 0.05; ** p < 0.01; *** p < 0.001

| State | Freq | iPF | iDur |
| --- | --- | --- | --- |
| AE | T=3.5. p=0.011 | T=-7.07. p<0.001 | T=-0.64. p=1 |
| QW | T=-4.1. p=0.004 | T=-7.44. p<0.001 | T=0.72. p=1 |
| SWS | T=-9.13. p<0.001 | T=-6.65. p<0.001 | T=4.24. p=0.004 |
| REM | T=0.08. p=0.104 | T=-5.30. p<0.001 | T=4.62. p=0.002 |

Supplementary Figure S3: Coherence spectra between LFP and Respiratory signals under CO_2_ condition, during the four brain states (AE active exploration, QW quiet waking, SWS slow-wave sleep, REM rapid eye movements sleep), in all structures, in the 0.5-10 Hz range. Results obtained from simultaneous recordings in each rat (grey lines) and averaged across animals (red lines). See sample size (number of rats) in Table 1 of main text.


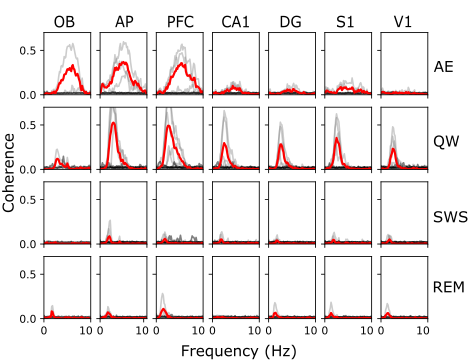


Supplementary Figure S4: Covariation between LFP and respiration frequencies. Covariation maps obtained from LFP signals recorded under ambient air (**A**) and CO_2_ (**B**) conditions in OB, AP, PFC, CA1, DG, S1, and V1. Y-axis represents LFP frequency and X-axis respiratory frequency. The map is normalized so that the total sum is 1 and point density is represented on a color scale ranging from blue to yellow as the point density increases. See Table 1 for samples size. AE active exploration, QW quiet waking, SWS slow-wave sleep, REM rapid eye movements sleep.


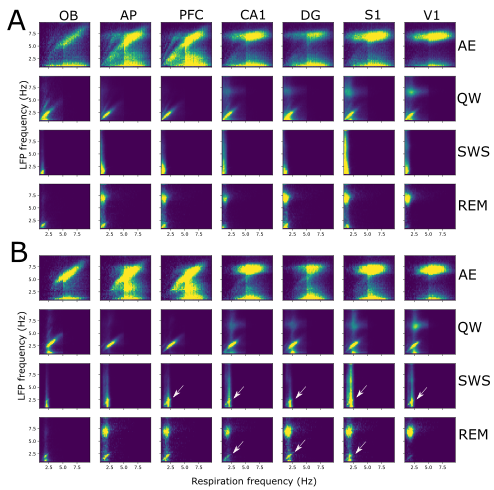

Supplement: Supplementary file 1 — Supplementary Information. [file 41598_2021_86525_MOESM1_ESM.docx]
